# The Development of Non-Enzymatic Glucose Biosensors Based on Electrochemically Prepared Polypyrrole–Chitosan–Titanium Dioxide Nanocomposite Films

**DOI:** 10.3390/nano7060129

**Published:** 2017-05-31

**Authors:** Ali M. A. Abdul Amir AL-Mokaram, Rosiyah Yahya, Mahnaz M. Abdi, Habibun Nabi Muhammad Ekramul Mahmud

**Affiliations:** 1 Department of Chemistry, Faculty of Science, University of Malaya, Kuala Lumpur 50603, Malaysia; rosiyah@um.edu.my; 2 Department of Chemistry, College of Science, Al-Mustansiriya University, Baghdad 10052, Iraq; 3 Department of Chemistry, Faculty of Science, University Putra Malaysia, Serdang 43400, Malaysia; mahnaz@upm.edu.my; 4 Institute of Tropical Forestry and Forest Products (INTROP), University Putra Malaysia, Serdang 43400, Malaysia

**Keywords:** nanomaterials, non-enzymatic glucose biosensors, nanocomposites, electrodeposition, titanium dioxide nanocomposite, X-ray photoelectron spectroscopy (XPS), electrochemical impedance spectroscopy (EIS)

## Abstract

The performance of a modified electrode of nanocomposite films consisting of polypyrrole–chitosan–titanium dioxide (Ppy-CS-TiO_2_) has been explored for the developing a non-enzymatic glucose biosensors. The synergy effect of TiO_2_ nanoparticles (NPs) and conducting polymer on the current responses of the electrode resulted in greater sensitivity. The incorporation of TiO_2_ NPs in the nanocomposite films was confirmed by X-ray photoelectron spectroscopy (XPS) spectra. FE-SEM and HR-TEM provided more evidence for the presence of TiO_2_ in the Ppy-CS structure. Glucose biosensing properties were determined by amperommetry and cyclic voltammetry (CV). The interfacial properties of nanocomposite electrodes were studied by electrochemical impedance spectroscopy (EIS). The developed biosensors showed good sensitivity over a linear range of 1–14 mM with a detection limit of 614 μM for glucose. The modified electrode with Ppy-CS nanocomposite also exhibited good selectivity and long-term stability with no interference effect. The Ppy-CS-TiO_2_ nanocomposites films presented high electron transfer kinetics. This work shows the role of nanomaterials in electrochemical biosensors and describes the process of their homogeneous distribution in composite films by a one-step electrochemical process, where all components are taken in a single solution in the electrochemical cell.

## 1. Introduction

Organic–inorganic nanocomposite materials have gained widespread attention because of the combined properties of organic and inorganic components discovered in these materials [1,2,3]. Conductive polymers and metal oxide nanocomposites with nanoscale dimensions are of special interest for improving the properties of sensors [4,5,6,7,8,9,10,11]. Metals oxides, such as copper oxide (CuO), titanium oxide (TiO_2_), and iron oxide (Fe_3_O_4_), are very often recognized as nano oxides in their native or modified forms for the oxidation of glucose [12,13,14]. Although glucose oxidase (GOx)-based enzymatic biosensors have been usually used for the detection of blood glucose since 1962 [15], thermal and chemical deformation can occur during fabrication, storage, and so on [16,17]. Therefore, non-enzymatic glucose sensors have become of great interest, particularly through the modification of the bare electrode surface with metal oxide nanoparticles (NPs). Modified nanoparticle electrodes provide a greater surface area, improving the electron transfer between the sensing reaction and the electrode [18,19,20,21].

Among the metal oxide NPs, titanium dioxide (TiO_2_) NPs have been exploited as a potential material for numerous applications because of their unique properties, including high surface area and high catalytic efficiency [22,23]. These properties work to enhance the interaction between biomolecules and the surfaces of electrodes because they afford a greater accessibility of the target molecules to the sensing area [24,25,26]. More recent efforts include using metal oxide and conducting polymers as non-enzymatic glucose sensors.

This paper describes the development of nanocomposite films made of polypyrrole–chitosan–titanium dioxide (Ppy-CS-TiO_2_) nanoparticles on ITO glass electrodes for the detection of glucose. The use of non-enzymatic glucose sensors involving TiO_2_ as the nanoparticle has not been reported before. Here, TiO_2_ NPs enhanced the sensitivity, stability, and selectivity of glucose biosensors via the prepared Ppy-CS-TiO_2_ electrode reported here.

## 2. Results and Discussion

**Figure 1:**
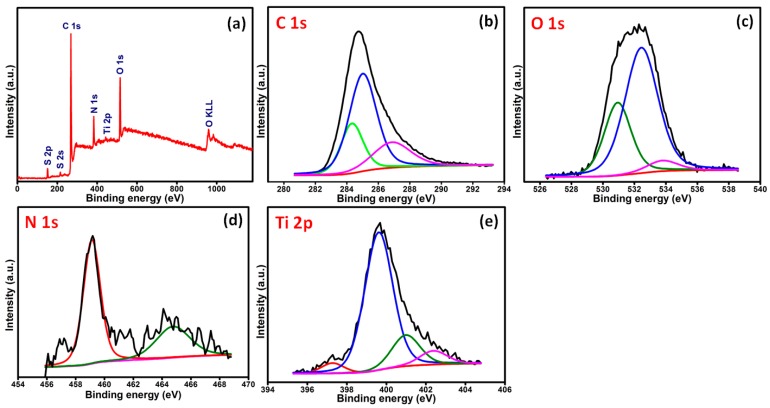
The XPS spectra of Ppy-CS-TiO_2_ nanocomposite films: (**a**) survey scan; (**b**) C 1s; (**c**) O 1s; (**d**) N 1s; and (**e**) Ti 2p narrow scans.

The presence of TiO_2_ nanoparticles in the nanocomposite films was confirmed by X-ray photoelectron spectroscopy (XPS). The XPS wide-scan spectra are presented in Figure 1a, and the XPS quantitative elemental analysis of narrow scans is presented in Figure 1b–e. For pyrrole, C ls is around 285 eV, N 1s is around 400 eV, and O ls is around 531 eV. The main carbon peak at 286.2 eV in chitosan corresponds to carbon bonded with both the hydroxyl group and nitrogen. In the carbon-related spectrum, two other significant peaks appeared and can be assigned to carbon–carbon single bonds 284.8 eV and carbonyl groups (approx. 287.8 eV), both of which are present in the chitosan structure. Moreover, the minor peak at the position of approximately 288.0 eV is noticeable. This peak can be observed as unreacted acidic acid hydroxyl groups (–COO) in Figure 1b [27]. The high-resolution spectrum, however, exposes at least two chemically different nitrogens. The stronger peak at 399.8 eV can be assigned to neutral –N–, whereas the higher binding energy peak at 401.0 eV is assigned to the oxidized –N^+^ moieties. These values are considered for the conducting of Ppy associated well with the value obtained electrochemically as seen in Figure 1d [28]. The O 1s peak for this nanocomposite appears with a split at 530.6 eV in Figure 1c and represents the lattice oxygen. It is shifted slightly compared to database values [29]. It is expected that this shift is caused by the particle diameter of less than 20 nm. Another O 1s peak shows at 532.5 eV and is due to the surface oxygen. In this case, the energy is higher compared to the bulk as there are open bonds. Since this material is of nanometer-size, it has a large specific surface area, which increases the surface-to-bulk oxygen ratio. It has been discussed that the ratio between the two peaks can be correlated to the surface area of the material [30]. In Figure 1e, the titanium atom shows two distinct peaks at 458.4 eV for the Ti2p_1/2_ and at 459.2 eV for the Ti2p_3/2_, showing clear evidence of the element present in the surface of the Ppy-CS-TiO_2_ nanocomposite films [31].

**Figure 2:**
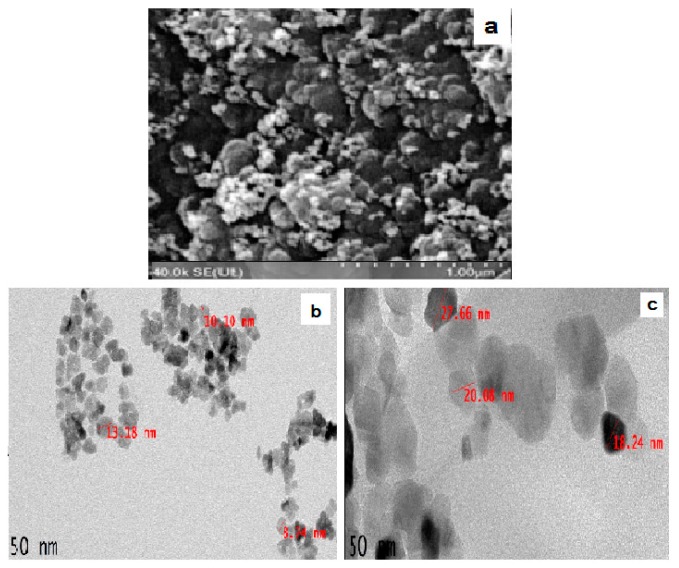
(**a**) FE-SEM micrograph of Ppy-CS-TiO_2_; (**b**) HR-TEM image of TiO_2_; (**c**) HR-TEM image Ppy-CS-TiO_2_ nanocomposite films.

FE-SEM micrographs confirmed the homogeneous distribution of TiO_2_ at the surface of the Ppy-CS-TiO_2_ nanocomposites films in Figure 2a, which shows some bright dots in colour of the nanoparticles of TiO_2_. The direct evidence for the incorporation of TiO_2_ nanoparticles in Py-CS-TiO_2_ nanocomposites was analyzed by HR-TEM. The size and dispersion of TiO_2_ nanoparticles and Ppy-CS-TiO_2_ nanocomposites were measured by HR-TEM in Figure 2b,c respectively. Approximately spherical agglomerated particles were observed for Ppy-CS-TiO_2_ nanocomposites with a size ranging from 18 to 27 nm. The size of the particles in the nanocomposite was higher than that of the TiO_2_ used (>20 nm). The TiO_2_ nanoparticles were found to be spherical in shape as seen in Figure 2b, and the particle size distribution of TiO_2_ nanoparticles was almost uniform. The Ppy-CS-TiO_2_ nanocomposites were found to have a typical incorporation of TiO_2_ into the composite as seen in Figure 2c. The dark areas represent the crystalline TiO_2_ nanoparticles, while the bright areas represent the amorphous CS, owing to the high electron density of the TiO_2_ nanoparticles. The interaction between CS and TiO_2_ enhanced the properties of CS-TiO_2_ nanocomposites.

**Figure 3:**
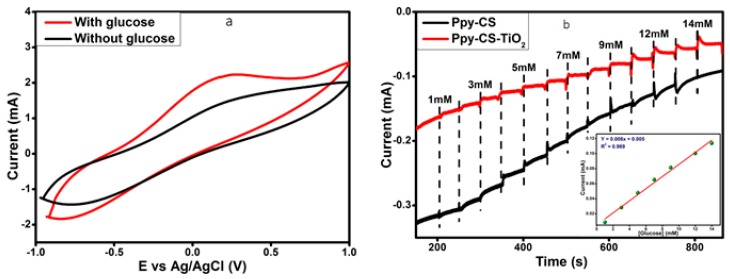
(**a**) CV responses of Ppy-CS-TiO_2_/ITO in 0.1 M NaOH electrolyte with 1 mM glucose and without glucose at the scan rate of 50 mV·s^−1^. (**b**) Amperometric responses to the successive addition of glucose concentration in 0.1 M NaOH solution at +0.13 V (vs. Ag/AgCl). The inset shows the steady-state calibration curve for the of Ppy-CS-TiO_2_ nanocomposite/ITO electrode.

The glucose sensing performances of Ppy-CS-TiO_2_ nanocomposite/ITO was carefully found by cyclic voltammetry (CV) in Figure 3a with and without glucose in alkaline media (0.1 M NaOH at pH 8.2) at a scan rate of 50 mV·s^−1^. In the presence of glucose, the peak currents for the Ppy-CS-TiO_2_ nanocomposite/ITO electrode were observed to be much higher than those without glucose. Thus, Ppy-CS-TiO_2_ nanocomposite/ITO acts as a good glucose sensor due to the glucose oxidation ability of the Ppy-CS-TiO_2_ nanocomposite.

On the basis of the reported glucose oxidation and the findings of this study, TiOOH-modified electrodes mediated the heterogeneous redox reactions, and the Ti(IV) species was subsequently regenerated, which mimicked the enzymatic oxidation reactions. The proposed mechanism of glucose oxidation on TiOOH is as follows [32,33,34,35,36,37]:

2TiO_2_ + C_6_H_12_O_6_ (Glucose) → 2TiOOH + C_6_H_10_O_6_ (Gluconolactone). (1)

2Ti(IV) + C_6_H_12_O_6_ (Glucose) → 2Ti(II) + C_6_H_10_O_6_ (Gluconolactone) + H_2_O_2_. (2)

C_6_H_10_O_6_ (Gluconolactone) + H_2_O → 2H^+^ + C_6_H_12_O_7_ (Gluconate). (3)

2Ti(II) → 2Ti(IV) + 2e^−^. (4)

Figure 3b shows typical steady-state amperometric responses of Ppy-CS-TiO_2_ nanocomposite/ITO and Ppy-CS/ITO with a successively increasing glucose concentration at an applied potential of +0.13 V (vs. Ag/AgCl). The current response of Ppy-CS-TiO_2_/ITO modified electrode toward glucose increased linearly, while the Ppy-CS/ITO presented a much lower response and only realized a slight increase with the addition of glucose into the cell. The results are consistent with those obtained from cyclic voltammograms. The calibration curve for Ppy-CS-TiO_2_ nanocomposite/ITO is presented in the inset of Figure 3b. The glucose sensors show a linear dependence on the glucose concentration with a dynamic range of 1–14 mM, with a correlation coefficient of 0.989, a sensitivity of 0.008 μA·cm^−2^·mM^−1^, and a low detection limit (LOD) of 614 μM of (S/N = 3). This novel Ppy-CS-TiO_2_ nanocomposite/ITO glucose sensor exhibits good sensitivity, a low detection limit, and a fast response time in less than 3 s. TiO_2_ nanoparticles in Ppy-CS-TiO_2_ showed a much higher current response compared to the electrode (Ppy-CS) without TiO_2_, as can be seen in Figure 3b for the amperometric responses to the successive addition of glucose.

**Figure 4:**
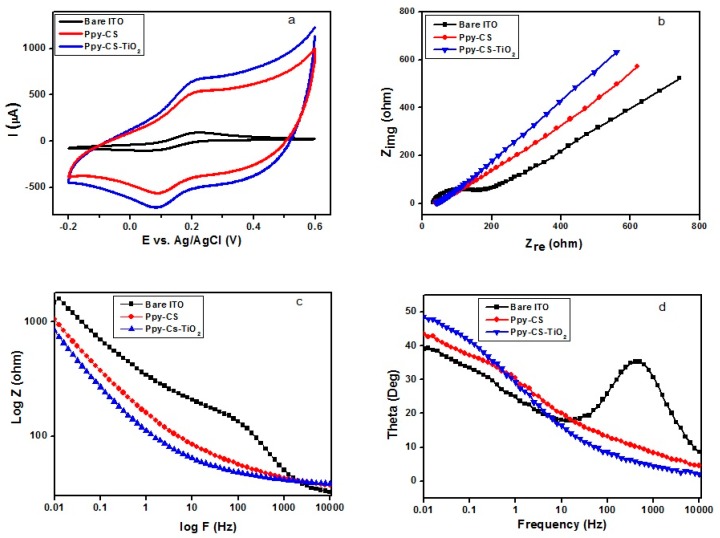
(**a**) Cyclic voltammograms obtained for bare ITO, Ppy-CS composite, and Ppy-CS-TiO_2_ nanocomposites; (**b**) Nyquist plots; (**c**) Bode impedance phase plots of log z; (**d**) Bode phase plots for 1 mM K_3_[Fe(CN)_6_] in 0.1 M KCl at a scan rate of 50 mV·s^−1^ vs. (Ag/AgCl).

Figure 4a describes the comparison of cyclic voltammetric responses obtained at the bare ITO, Ppy-CS composite and Ppy-CS-TiO_2_ nanocomposites for 1 mM K_3_[Fe(CN)_6_] in 0.1 M KCl at a scan rate of 50 mV s^−1^. The bare ITO electrode shows a reversible voltammetric characteristic for the one electron redox process of [Fe(CN)_6_]^3−/4−^ at a scan rate of 50 mV·s^−1^. The Ppy-CS composite and Ppy-CS-TiO_2_ nanocomposite electrodes show in Figure 4a enhanced redox peak currents with a peak-to-peak separation, when compared to bare ITO. The high conductivity of Ppy facilitated the electron transfer and presented a higher peak current of oxidation and reduction peak values (480 µA and −500 µA) for the Ppy-CS composite electrode and likewise for the nanocomposite electrode (580 µA and −580 µA). It was noticeably found that the nanocomposite electrode performs as a new electrode surface; this is due to the effect of both TiO_2_ and Ppy, which increased the electrocatalytic activity and appeared as a good electrical communicator with the original electrode surface.

The interfacial properties of nanocomposite electrodes were studied by electrochemical impedance spectroscopy (EIS) in Figure 4b. The Nyquist diagram of the complex impedance shows that the bare ITO electrode has a roughly semicircle-shaped Nyquist plot with a large diameter, which recommends the hindrance to the electron-transfer kinetics at the electrode. For Ppy-CS-TiO_2_ nanocomposites, no semi-circle was observed, which shows the higher electron transfer kinetics. Additionally, the composite and nanocomposite electrodes presented only the linear portion at lower frequencies. This semicircle compared to the other electrodes, which is a result of the large charge-transfer resistance (R_ct_) at the electrode/electrolyte interface due to the poor electron transfer kinetics. It can obviously be seen that (R_ct_) decreased for the Ppy-CS composite and Ppy-CS-TiO_2_ nanocomposite electrodes, which can be attributed to the presence of high conductive Ppy and catalytically active TiO_2_ nanoparticles on the electrode surface. The diffusion-limited process was much more facilitated at the nanocomposite electrodes owing to the conducting properties of Ppy and the large surface area of TiO_2_ in the nanocomposites.

The conducting nature of Ppy in both the composite and nanocomposite electrode facilitates the peak shifting in the Bode plot showed in Figure 4c. The Bode impedance plot of the nanocomposite electrode, compared to the composite electrodes, presented a lower log*Z* value in a low frequency range of 1–100 Hz.

In Figure 4d, the Bode-phase plots of the nanocomposite electrodes were collected in the frequency range of 0.01–10,000 Hz. The phase peaks appeared at a frequency range of 100–10,000 Hz, which corresponds to the charge-transfer resistance of the nanocomposite electrodes. The shifting of peaks toward the low frequency region of 1–0.01 Hz for composite and nanocomposite electrodes indicates the fast electron-transfer behavior of the nanocomposites. A perfect linear portion was observed at lower frequencies for the nanocomposite electrode compared to the other electrodes. These results indicate that the Ppy-CS-TiO_2_ nanocomposite was successfully designed and it facilitated a diffusion-limited process at the electrode-solution interface.

**Figure 5:**
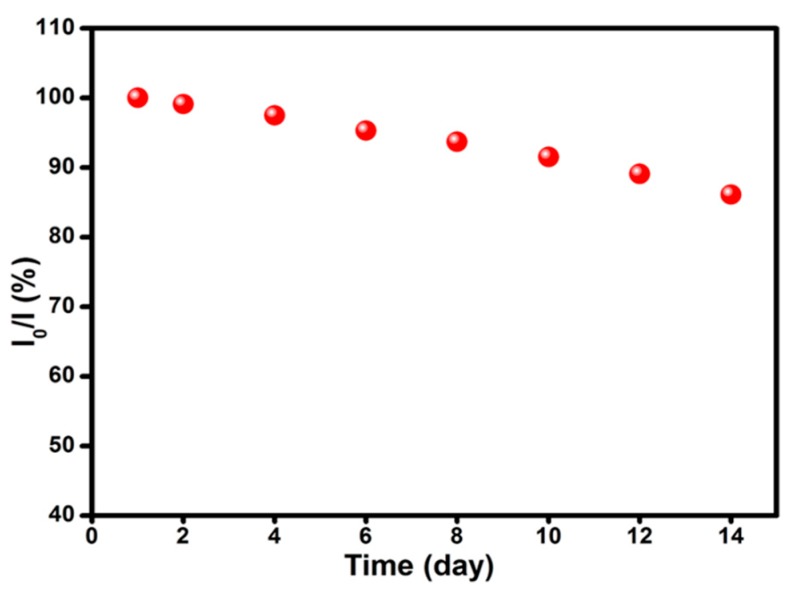
Stability of the sensor stored at ambient conditions over 14 days in 0.1 M NaOH glucose at potential of 0.13 V (vs. Ag/AgCl).

The stability of the developed sensor was investigated by measuring its current response for glucose for 14 days. The prepared Ppy-CS-TiO_2_ nanocomposite films on ITO was used to record the amparometric response for 1 mM glucose with a frequency of 2 days. Figure 5 represented the stability of the Ppy-CS-TiO_2_/ITO modified electrode over 14 days. I_0_ is the current response of the fresh sensor, and I_14_ is the current response after 14 days storage, as shown in Figure 5. During the first six days, the current response did not change in a noticeable way. However, on the 14th day (the last day), the current response still remained above 88% of its initial response, revealing the excellent stability of the non-enzymatic glucose sensor.

**Figure 6:**
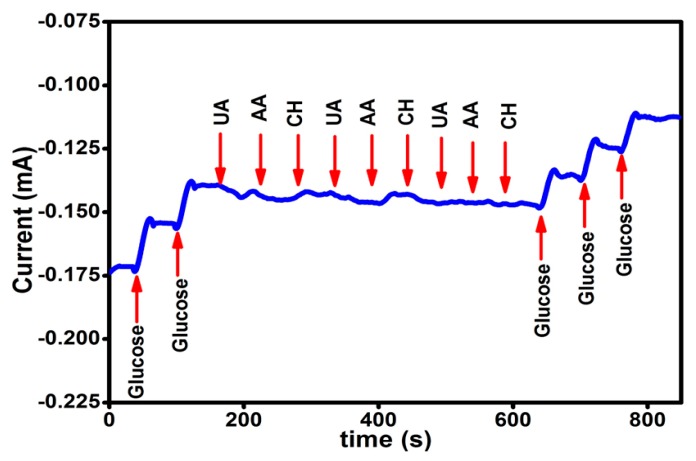
Amperometric responses obtained at successive addition of glucose and each of UA, AA, and CH in 0.1 M NaOH solution at +0.13 V (vs. Ag/AgCl) with regular intervals of 50 s.

The selectivity of the Ppy-CS-TiO_2_ nanocomposite/ITO for the detection of glucose was examined by injecting three different interfering biomolecules, namely, uric acid (UA), ascorbic acid (AA), and cholesterol (CH) in the evenly stirred 0.1 M NaOH solution containing glucose. Figure 6 depicts the recorded amperometric responses for the consecutive additions of glucose and the interfering biomolecules. The first, successive addition of 1 mM glucose were used to record the current response for two glucose injections after every 50 s. The clear response was observed towards glucose detection while no response was detected after consecutive addition of 0.1 mM UA, AA, and CH in the same stirred solution. In order to check selectivity another three-successive glucose addition were done which caused increasing in the current response. It was found that the addition of interfering biomolecules did not contribute significant changes to current response contrary to glucose solution, which represented a high response for each injection. These results revealed that the present sensor possesses good selectivity and sensitivity towards the detection of glucose, even in the presence of common physiological interfering biomolecules.

## 3. Materials and Methods

Chitosan was purchased from purchased from (ACROS Organics, Morris Plains, NJ, USA). TiO_2_ NPs >20 nm, d(+)glucose sodium, para toluene sulfonate (*p*-TS), AA, UA, and CH were purchased from (Sigma-Aldrich, St. Louis, MO, USA). Acetic acid, sodium hydroxide, and freshly distilled pyrrole 99% were provided from (Merck, Selangor, Malaysia). The indium tin oxide (ITO) glass electrode was purchased from (Fluka, Kozu, Chuo Ward, Osaka, Japan). A stock solution of d(+)glucose was prepared (1 M) and left overnight to study the sensing performance of the modified electrode. A stock solution of NaOH (0.1 M) was prepared using distilled water. A stock solution was prepared containing potassium ferricyanide (1 × 10^−3^) M K_3_[Fe(CN)_6_] in potassium chloride (0.1) M KCl.

### 3.1. Instrument

Field emission scanning electron microscope (FE-SEM) from (Hitachi Brand, Model SU 8220, Tokyo, Japan) and high-resolution transmission electron microscope (HR-TEM) from (Tecnai model G2-F20, Twin manufacture, FEI, Hillsboro, Oregon, OR, USA) were used for the investigation of the morphology and structural properties of the deposited nanocomposite films. The presence of the nanoparticles in the prepared films was determined by X-ray photoelectron spectroscopy (XPS) brand (ULVAC-PHI Quantera II) from (Ulvac-PHI, INC, Chigasaki, Kanagawa, Japan). All electrochemical measurements were performed using a computerized potentiostat instrument made by (Digi-ivy, Inc., Austin, TX, USA). A conventional three-electrode cell system comprised of an ITO glass electrode, graphite rode, and Ag/AgCl were used as a working electrode, counter electrode, and reference electrode, respectively.

### 3.2. Preparation of Ppy-CS-TiO_2_ NP/ITO Nanocomposite Films

TiO_2_ NPs were dispersed into 25 mL of CS (50 mg/mL) solution under continuous stirring at room temperature followed by ultrasonication for 2 h to obtain a viscous solution of CS with uniformly dispersed TiO_2_ NPs. Later, a certain amount of pyrrole and *p*-TS as a dopant were added to the CS and TiO_2_ dispersion and stirred for 5 min. The prepared dispersion of Ppy-CS-TiO_2_ was used for the electrochemical deposition of the film on the ITO glass electrode by cyclic voltammetry scanning ranging from −1 to +1.2 V (vs. Ag/AgCl) with a scan rate of 50 mV/s using the three-electrode cell system. The Ppy-CS-TiO_2_ nanocomposite films were then washed repeatedly with distilled water to remove any unbound particles and later dried at room temperature. The prepared films were characterized and subjected to electrochemical oxidation of glucose.

### 3.3. Mechanism for the Formation of Ppy-CS-TiO_2_ on ITO

**Figure 7:**
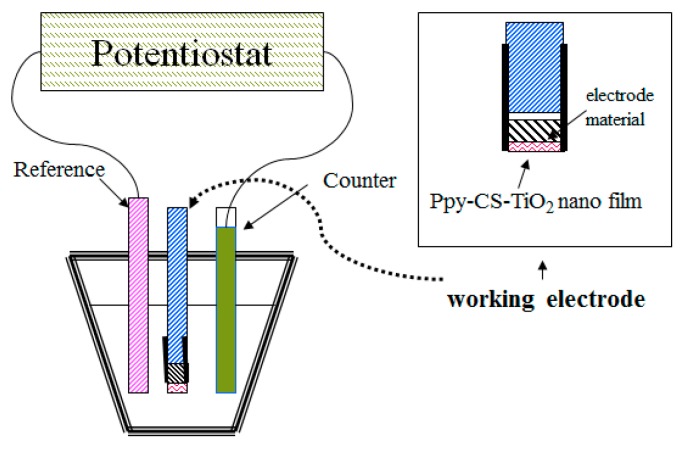
The electrochemical cell of Ppy-CS-TiO_2_ film preparation.

In this study, cyclic voltammetry was used for the electrochemical deposition of conducting polymer nanocomposite films on the ITO glass electrode, where all of the components, including the monomer, the dopant, chitosan, and the nanoparticle, were dissolved in the solvent in the electrochemical cell as seen in Figure 7.

The performance of the developed sensor was compared with reported results in the literature displayed in Table 1.

**Figure 8:**
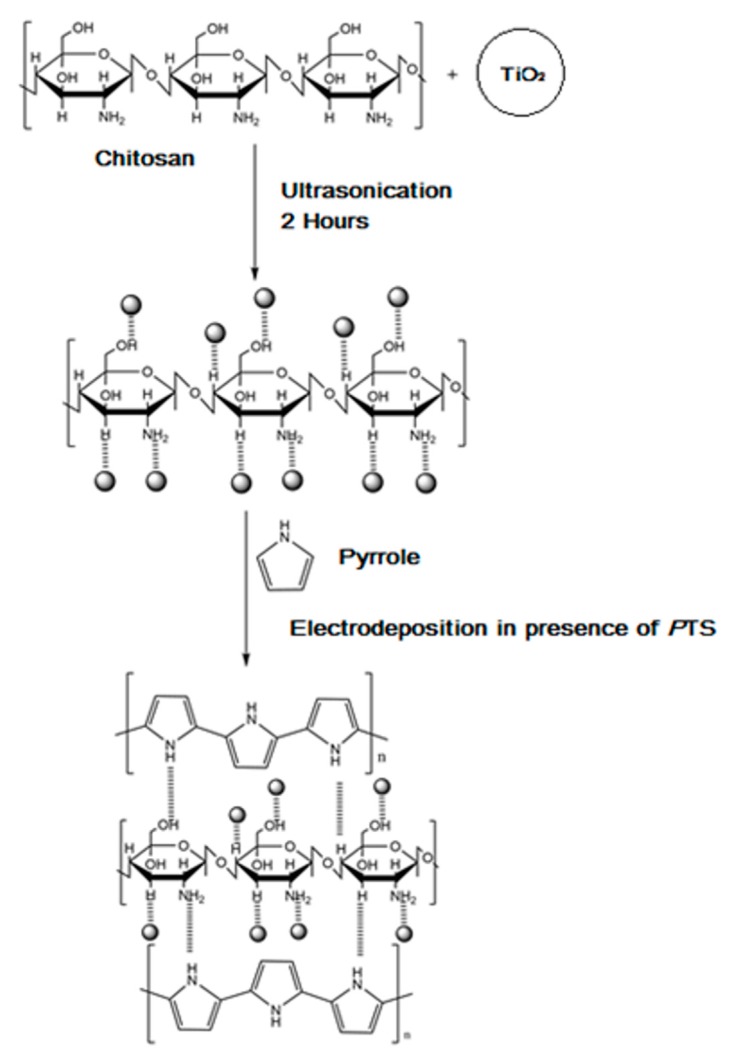
The mechanism of Ppy-CS-TiO_2_ film electrodeposition.

During this one-step, electrochemical formation of CS-Ppy-TiO_2_ nanocomposite films, polypyrrole is linked with CS-TiO_2_ through hydrogen bonding and titanium–nitrogen ligand formation. The CS-TiO_2_ is linked with hydrogen bonding during ultra-sonication in the preparatory stage as shown in Figure 8.

## 4. Conclusions

In summary, the preparation of Ppy-CS-TiO_2_ nanocomposite films on an ITO glass electrode was successful as non-enzymatic glucose biosensors. The electrochemically prepared nanocomposite films (Ppy-CS-TiO_2_) exhibited an excellent electrocatalysis towards glucose oxidation in alkaline media. TiO_2_ as a nanomaterial played a vital role in glucose oxidation together with polypyrrole and chitosan. The nanocomposite films showed a low detection limit, a wide linear range, a fast response time, and a high current for glucose oxidation with good stability. EIS showed the lowest charge transfer resistance for the prepared (Ppy-CS-TiO_2_) nanocomposite films. As a glucose sensor, the prepared (Ppy-CS-TiO_2_) nanocomposite electrode is immune to other biomolecules such as uric acid, ascorbic acid, and cholesterol. Considering this low-cost, facile, and controllable method for the preparation of (Ppy-CS-TiO_2_) nanocomposite films and the improved electrocatalytic activity toward glucose oxidation, the future of this non-enzymatic glucose sensor looks bright.

## Figures and Tables

**Table 1:** Performance parameters obtained from different electrodes based on non-enzymatic glucose biosensors.

Electrode Material	Technique Methods	Electrolyte	Linear Range (mM)	Detection Limit (μM)	Reference
Au/Nafion	Amperometry	0.1 M NaOH	5.0–60	1200	[38]
Layer-by-layer Au NPs/Au E	Nil	0.1 M NaOH	Up to 8	500	[39]
Au NPs/chitosan/GCE	Nil	PBS	0.4–10.7	370	[40]
GCE/GNPs/PpyNFs	Amperometry	0.1 M NaOH	0.2–13	-	[41]
Ppy-CS-Fe_3_O_4_NP/ITO	Amperometry	0.1 M NaOH	1–16	234	[42]
TiO_2_/LAC	Amperometry	0.1 M NaOH	3.75–150	3.75	[43]
Ppy-CS-TiO_2_NP/ITO	Amperometry	0.1 M NaOH	1–14	614	This work

NP: nanoparticles; E: electrode; GCE: glassy carbon electrode; PpyNFs: polypyrrole nanofibers; GNPs: gold nanoparticles; LAC: Laccase.
